# Initial skin necrosis presentation at emergency room was associated with fulminant clinical course and mortality in patients with *Vibrio* necrotizing fasciitis

**DOI:** 10.1038/s41598-023-45854-1

**Published:** 2023-10-27

**Authors:** Chun-Yuan Hsiao, Tsung-Yu Huang, Li-Yun Teng, Hung-Yen Chen, Cheng-Ting Hsiao, Yao-Hung Tsai, Shu-Fang Kuo

**Affiliations:** 1 Department of Orthopaedic Surgery, Chia-Yi Chang Gung Memorial Hospital, No. 8, West Sec, Chia-Pu Road, Putz City, Chiayi County, 61363 Taiwan, Republic of China; 2 College of Medicine, Chang Gung University at Taoyuan, Taoyuan City, Taiwan, Republic of China; 3 Division of Infectious Diseases, Department of Internal Medicine, Chia-Yi Chang Gung Memorial Hospital, Chiayi County, Taiwan, Republic of China; 4 Microbiology Research and Treatment Center, Chia-Yi Chang Gung Memorial Hospital, Chiayi County, Taiwan, Republic of China; 5 Department of Physical Medicine and Rehabilitation, Taichung Tzu Chi Hospital, Taichung City, Taiwan; 6 Department of Emergency Medicine, Chia-Yi Chang Gung Memorial Hospital, Chiayi County, Taiwan, Republic of China; 7 Departments of Laboratory Medicine, Chia-Yi Chang Gung Memorial Hospital, Chiayi County, 61363 Taiwan

**Keywords:** Diseases, Risk factors

## Abstract

Necrotizing fasciitis (NF) is a life-threatening infection. Skin necrosis is an important skin sign of NF. The purposes of this study was to investigate the initial skin conditions of *Vibrio* NF patients between emergency room (ER) to preoperative status, to compare the clinical and laboratory risk indicators of the skin necrosis group and non-skin necrosis group when they arrived at ER, and to evaluate whether initial cutaneous necrosis related to fulminant course and higher fatalities. From 2015 to 2019, seventy-two *Vibrio* NF patients with surgical confirmation were enrolled. We identified 25 patients for inclusion in the skin necrosis group and 47 patients for inclusion in the non-skin necrosis group due to the appearance of skin lesion at ER. Seven patients died, resulting in a mortality rate of 9.7%. Six patients of skin necrosis group and one patient of non-skin necrosis group died, which revealed the skin necrosis group had a significantly higher mortality rate than the non-skin necrosis group. All the patients in the skin necrosis group and 30 patients of non-skin necrosis group developed serous or hemorrhagic bullous lesions before operation (*p* = 0.0003). The skin necrosis group had a significantly higher incidence of APACHE score, postoperative intubation, Intensive care unit stay, septic shock, leukopenia, higher counts of banded leukocytes, elevated C-reactive protein (CRP), and lower serum albumin level. *Vibrio* NF patients presenting skin necrosis at ER were significantly associated with fulminant clinical courses and higher mortality. Physicians should alert the appearance of skin necrosis at ER to early suspect NF and treat aggressively by those clinical and laboratory risk indicators, such as elevated APACHE score, shock, leukopenia, higher banded leukocytes, elevated CRP, and hypoalbuminia.

## Introduction

Necrotizing fasciitis (NF) is a life-threatening skin and soft tissue infection with rapid and progressive clinical courses which presents a surgical emergency^1–3^. Early stages of NF were difficult to differentiate at initial onset with cellulitis, abscesses and erysipelas because they had similar skin lesions, progressive erythematous change and pain out of proportion in the emergency room (ER), which leads to delayed or missed diagnosis^4–9^. The cutaneous features of NF were defined three stages, and the cutaneous presentations in stage 2 and 3, such as serous-filled bullae and hemorrhagic bullae, played a crucial role in the diagnosis of NF^9–12^. Bullae formation indicated critical skin ischemia and was an important diagnostic clue of NF; however, not all NF patients presented bullae formation in clinical settings at ER.

Our previous studies had demonstrated hemorrhagic bullous lesions could be effectively used to differentiate NF from cellulitis at initial onset, and they were significantly associated with the gram-negative bacteria infection and mortality^9,12–16^. We also found some admitted cellulitis patients with the skin signs of erythema, swelling, warmth and tenderness at ER, revealed progressive edematous pain, hemorrhagic bullous lesions and septic conditions at the time of consultation in the ward; eventually, NF was diagnosed few hours or days after admission^9,12,13^. Our institution is situated on the western coast of southern Taiwan, and most of the residents’ occupations were fishermen or farmers, and *Vibrio* species was the leading causative pathogen of NF and related fatality in our institution^9,12–20^. Although β-hemolytic *Streptococcus*, *Staphylococcus aureus* and polymicrobial pathogens were most commonly reported to cause NF in the literatures, they did not often present hemorrhagic bullous skin lesion in our previous reports^9,12,13,16^.

Skin necrosis is a type of tissue death caused by lack of blood supply and subsequent decay of body tissues caused by infection or vascular thrombosis; usually, it shows up as a purplish, bluish or black skin coloration, detachment of local skin, and gangrene^7,11,21^. We had found the gram-negative NF had a significant higher prevalence of hemorrhagic bullous formation than gram-positive NF, and *Vibrio* species revealed more clinical fulminant course and bullous skin progression than other pathogens did^9,12–20^. Thus, we sought to identify skin lesions in *Vibrio* patients, characterized by necrotic change, purple skin discoloration, or skin erosion, which could be another alert signs for suspecting NF for physicians at ER.

The purposes of this study was to investigate the initial skin conditions of *Vibrio* NF patients between ER to preoperative status, to compare the clinical and laboratory risk indicators of the skin necrosis group and non-skin necrosis group when they arrived at ER, and to evaluate whether initial cutaneous necrosis related to fulminant course and higher fatalities.

## Method

### Patient selection and data extraction

We performed a retrospective cohort study evaluating 299 patients with diagnosis of necrotizing fasciitis of limbs who were admitted to ER and underwent surgical intervention at Chia-Yi Chang Gung Memorial Hospital from January 2015 to December 2019. The retrospective study was conducted in accordance with the ethical standards of the institutional and national research committee and the guidelines of the Declaration of Helsinki, and was approved by the Institutional Review Board (202001656B0 and 201801530B1B0) of Chang Gung Medical Foundation and Chang Gung University. Due to the nature of this retrospective study and the preserved anonymity of patients, a waiver of informed consent was obtained from Chang Gung Medical Foundation/IRB.

**Figure 1:**
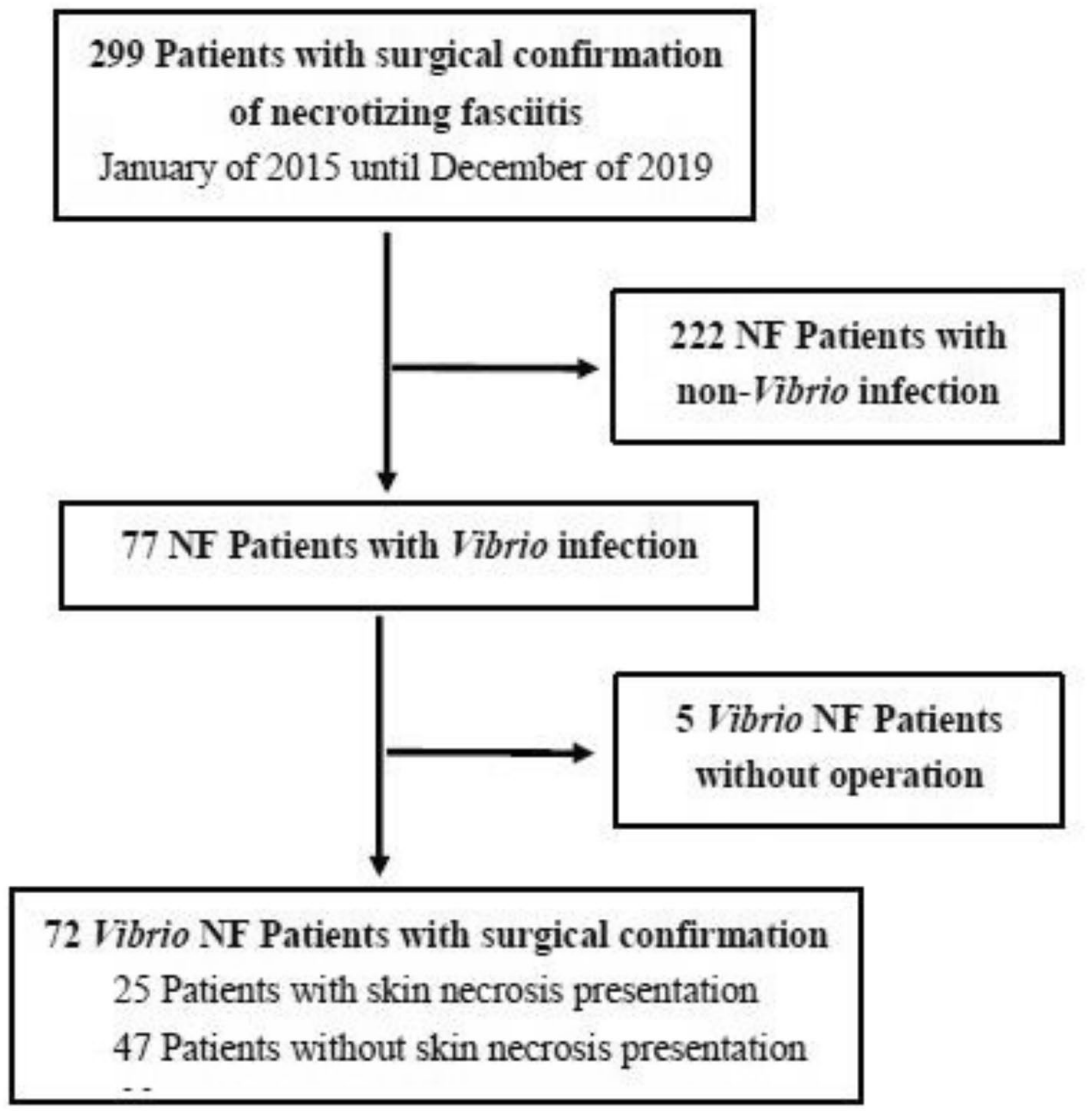
Flow chart of patient inclusion.

Routinely blood cultures and pictures of skin condition were collected at ER. We took pictures of skin lesions before and after surgery, and obtained the wound cultures with sterile cultrate swabs during surgery. The cultured specimens of patients were confirmed by microbiologic evaluation few days after surgery. The inclusion criteria was as followed: (1) NF was confirmed by surgery when necrotic tissue was observed macroscopically and histopathologic confirmation by the presence of necrotic tissues at the time of excisional debridement, fasciotomy, or immediate limb amputation, (2) *Vibrio* species infection that was detected in wound or blood culture. A total of 72 monomicrobial *Vibrio* NF patients were included in this study (Fig. 1).

### Clinical assessment

Patient characteristics including age, sex, site if infection, antibiotics usage, Acute Physiology and Chronic Health Evaluation (APACHE) II scores upon admission, use of postoperative intubation, ICU stay, length of hospitalization, amputation, as well as mortality rates were documented after reviewing the medical records. Furthermore, laboratory examinations and clinical presentations were collected upon arrival at ER.

The NF patients with skin necrosis mentioned in medical records and pictures taken at ER, such as purplish, bluish or black skin coloration, detachment of local skin, gangrene, skin breakdown, and foul-smelling discharge leaking, were classified into skin necrosis group. Cutaneous manifestations of hemorrhagic or serous bullae formation were also recorded before preoperative status by taking pictures in the operation room (OR). We identified 25 patients for inclusion in the skin necrosis group and 47 patients for inclusion in the non-skin necrosis group who met the criterias.

### Statistical analysis

Statistical analyses were performed with the use of SPSS version 18.0 software (SPSS, Inc., Chicago, IL, USA). Continuous data were presented as means ± SD and categorical variables were expressed as absolute number or percentages. We used the two-tailed* t*-test for continuous variables and the Fisher exact test for categorical variables to examine significant relationships between risk factors and outcomes between skin necrosis and non-skin necrosis groups. A value of *p* < 0.05 (two tailed) was considered significant.

### Ethics approval and consent to participate

The study was conducted according to the guidelines of the Declaration of Helsinki, and approved by the Institutional Review Board (202001656B0 and 201801530B1B0) of Chang Gung Medical Foundation and Chang Gung University.

## Results

Seven patients died, resulting in a mortality rate of 9.7%. Six patients had received amputation with an amputation rate of 8.3%. Forty patients had upper limbs and 32 patients had lower limbs involvement. Six patients of skin necrosis group (24%) and one patients of non-skin necrosis group (2.1%) died, which revealed that the skin necrosis group had a significantly higher mortality rate than the non-skin necrosis group (*p* = 0.005) (Table 1).

**Table 1:** Characteristics comparison between the skin necrosis group and non-skin necrosis group in patients with necrotizing fasciitis at emergency room.

Variable	Skin necrosis group (N = 25)	Non-skin necrosis group (N = 47)	*p*-value
Age (years)	70.4 ± 15.8	66.7 ± 14.4	0.321
Gender			
Male	19 (76%)	33 (70.2%)	0.783
Female	6 (24%)	14 (29.8%)	
Seawater and seafood contact	20 (80%)	38 (80.8%)	1.000
APACHE II score	14.21 ± 4.43	11.55 ± 5.54	0.043*
Postoperative intubation	11 (44%)	7 (14.9%)	0.01*
ICU stay	20 (80%)	20 (42.5%)	0.003*
Site of involved limb			
Upper extremity	9 (36%)	31 (65.9%)	
Lower extremity	16 (64%)	16 (34.1%)	
Systemic clinical presentation			
Fever (Temperature ≥ 38.5℃)	6 (24%)	12 (25.5%)	1.000
Tachycardia (Heart rate ≥ 100)	13 (45.5%)	27 (57.4%)	0.803
Tachypnea (Respiration rate ≥ 20)	13 (45.5%)	24 (39.3%)	1.000
Shock (SBP < 90 mmHg)	16 (64%)	10 (21.3%)	0.006*
Bacteremia	19 (76%)	32 (68%)	0.590
Cutaneous clinical presentation			
Preoperative bullae presentation			
Hemorrhagic or serous bullae	25 (100%)	32 (68.1%)	0.0007*
Non bullous formation	0	15 (31.9%)	
Clinical outcomes			
Mortality	6 (24%)	1 (2.1%)	0.005*
Amputation	2 (8%)	4 (8.5%)	0.625
Hospital stay (days)	39.8 ± 21.1	31.9 ± 17.9	0.193

*Mean *p* < 0.05 and the difference was significant.

Broad-spectrum antibiotics were initially administered to patients with suspecting *Vibrio* NF at ER: ceftriaxone and doxycycline in 52 cases, ceftriaxone alone in 12 cases, ceftriaxone and vancomycin in 4 case, and ceftriaxone with teicoplanin in 4 case. These antibiotics were continued after surgery under the supervision of infectious doctors. *Vibrio vulnificus* were the most dominant pathogen, followed by *Vibrio cholerae* non-O1 and *Vibrio parahaemolyticus*. All *Vibrio* isolates were susceptible to ceftazidime, ceftriaxone, levofloxacin, and tetracycline.

**Figure 2:**
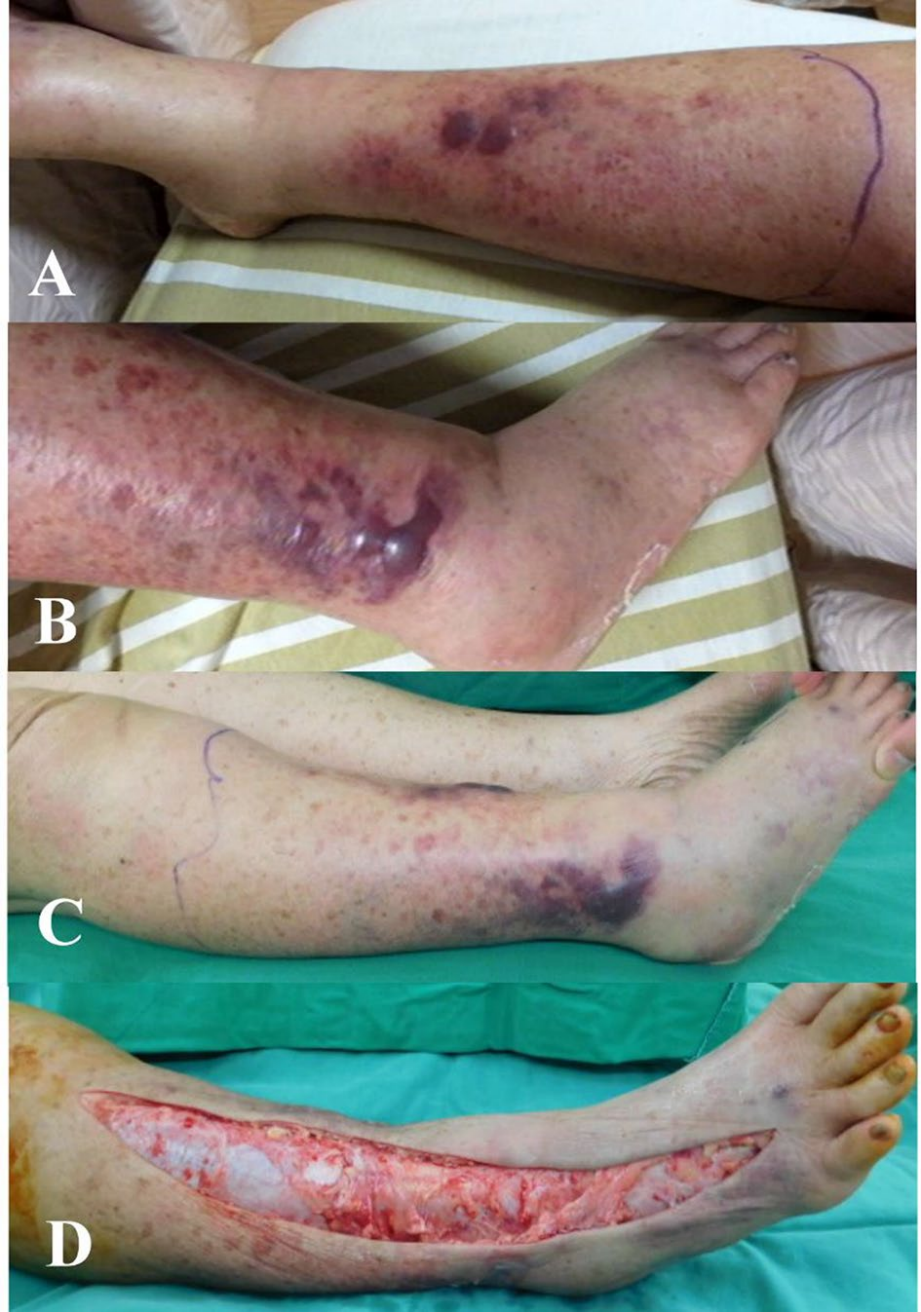
Skin necrosis group: A 77 year-old female with a history of renal cell carcinoma and chronic renal insufficiency had right low leg pain and swelling for 2 days due to contact with seawater. (**A**,**B**) Photographs of right lower leg revealed severe patchy purpura and hemorrhagic bullae in the emergency room. (**C**) She was sent to operation room four hours later, and the skin showed progressive erythematous change. (**D**) After emergency fasciotomy, the blood and wound cultures confirmed the presence of *Vibrio vulnificus*. She had received skin graft on the 33rd day after fasciotomy and discharged on 47th day.

**Figure 3:**
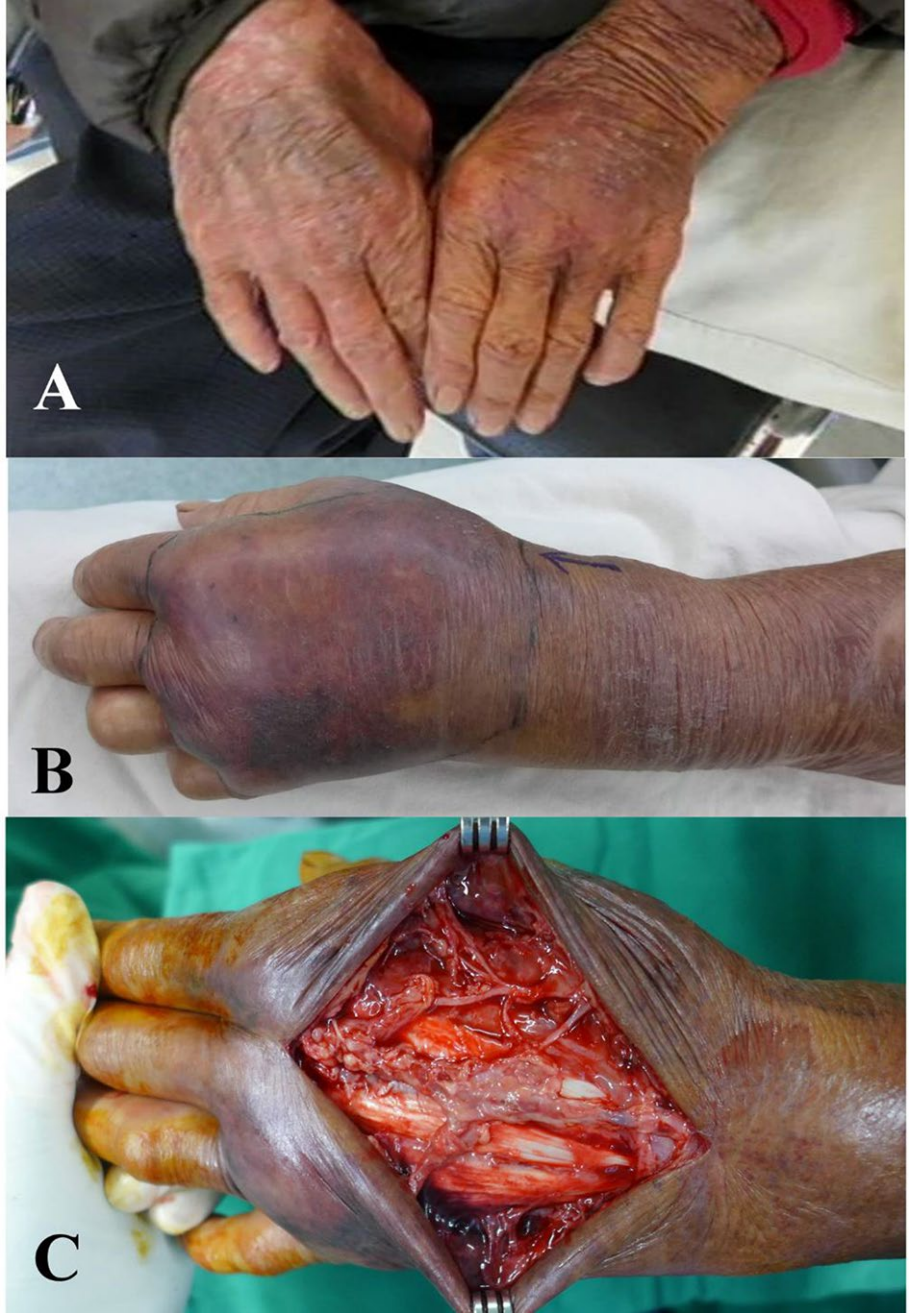
Non-skin necrosis group: A 80 year-old male with a history of liver cirrhosis, hepatitis and heart disease had left dorsal hand swelling and tender for 1 day after handling fish. (**A**) Photographs of left hand showed mild swelling and erythema in the emergency room, and he was treated as cellulitis. (**B**) Six hours later, his left hand revealed progressive swelling and edematous change, and necrotizing fasciitis was diagnosed. The involved hand had showed hemorrhagic bullae and skin gangrene. (**C**) After emergency fasciotomy, the blood and wound cultures confirmed the presence of *Vibrio vulnificus*.

**Figure 4:**
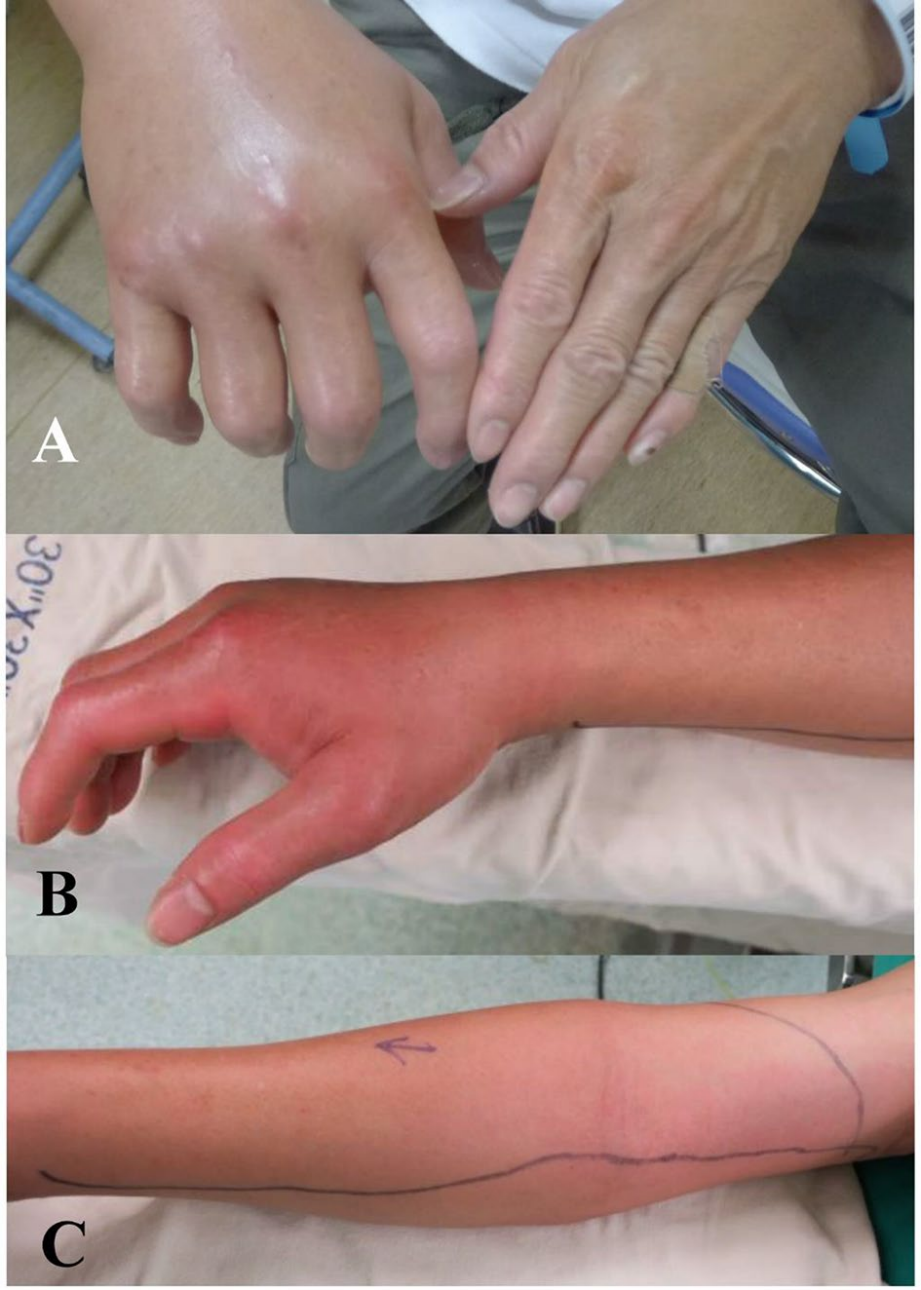
Non-skin necrosis group: A 49 year-old male with a history of hepatitis B had right hand edema and pain for 1 day due to fish sting. (**A**) Photographs of right hand showed severe swelling in the emergency room, and he was admitted for antibiotics treatment with the diagnosis of cellulitis. (**B**) (**C**) Seven hours later, the right hand revealed progressive erythematous change without hemorrhagic or serous bullae, and necrotizing fasciitis was suspected. After emergency fasciotomy, the blood culture confirmed *Vibrio vulnificus*.

Age, sex, seawater and seafood contact, fever, tachycardia, bacteremia, amputation rate and hospital stay did not differ significantly between skin necrosis group and non-skin necrosis group. The skin necrosis group had a significantly higher incidence of elevated APACHE score, postoperative intubation, ICU stay, and systolic blood pressure (SBP) < 90 mmHg than non-skin necrosis group. all the patients in the skin necrosis group had revealed progressive skin gangrene change and hemorrhage bullae formation before operation (Fig. 2). Thirty-two patients (68.1%) of non-skin necrosis group developed hemorrhagic or serous bullous skin lesion, and 15 patients showed intact skin without bullous lesion preoperatively in the OR (Figs. 3 and 4). This indicated initial skin necrosis presentation at ER had significantly developed to NF (*p* = 0.0007).

We found that the patients of skin necrosis group had a significantly higher incidence of WBC counts < 3500 cells/mm^3^ (normal ranges, 3500 to 11,000 cells/mm^3^), higher counts of banded leukocytes, elevated C-reactive protein (CRP), and lower serum albumin level than those patients in non-skin necrosis group (Table 2).

**Table 2:** *Vibrio* Species and laboratory data comparison between the skin necrosis group and non-skin necrosis group in NF patients at emergency room.

	Skin necrosis group (N = 25)	Non-skin necrosis group (N = 47)	*p*-value
Pathogen			
* Vibrio vulnificus*	24	45	
* Vibrio cholerae* non-O1	1	1	
* Vibrio parahaemolyticus*	0	1	
White blood cells counts (cells/mm^3^)	12,048 ± 6824	15,695 ± 9920	0.105
Leukocytosis (WBC ≥ 11,000)	11 (44%)	32 (68.1%)	0.076
Leukopenia (WBC < 3500)	3 (12%)	0 (1.6%)	0.038*
Band form (%)	8.38 ± 9.33	3.69 ± 6.15	0.0126*
0	9	26	0.142
> 0	16	21	
Platelet counts (per mm^3^)	140,720 ± 71,849	155,468 ± 59,874	0.356
≤ 150,000	14	22	0.621
> 150,000	11	25	
Hemoglobulin (g/dL)	169,731 ± 104,854	186,541 ± 84,099	1.000
< 10	2	4	1.000
≥ 10	23	43	
Lactate (mmol/L)	38.8 ± 31.2	28.3 ± 16.7	0.065
C-reactive protein (mg/L)	98.7 ± 123.1	45.1 ± 62.6	0.016*
Lymphocyte count (cells/mm3)	1011.6 ± 796.3	1099.1 ± 707.9	0.634
Albumin level (g/dL)	3.28 ± 0.79	3.64 ± 0.42	0.0135*
< 3	11	5	0.002*
≥ 3	14	42	

*Mean *p* < 0.05 and the difference was significant.

## Discussion

NF is a surgical emergency due to its high mortality rate, and the most common early signs of NF are erythema, local warmth, skin sclerosis, and edema^1–9^. Kiat et al. had reviewed that the most reliable cutaneous signs were swelling (79–80.8%) and erythema (69.6–70.7%), but they could delay the diagnosis of NF because the skin lesions of involved limbs presented as similar as cellulitis at ER^8,9,11^. Serous or hemorrhagic bullae formation was considered the key sign to distinguish early and late stages of NF; however, not all patients with NF could demonstrate bullae formation initially^9–15,22,23^.

*Vibrio* species are the most frequent causative organisms of monomicrobial NF, and were proved to occur more rapidly progressive and fulminant clinical courses than other pathogens in our institution^12–20,22^. According to our report, 58 patients (80.6%) with *Vibrio* infections had a recent history of contact with seawater or raw seafood, which we could early suspect *Vibrio* NF at ER, and ceftriaxone with/without other appropriate antibiotics under the supervision of infectious doctors appears to have a clinical effectiveness for the treatment of *Vibrio* NF^12–20^. Hemorrhagic bullous presentation has become an important clinical sign for suspecting NF and surgical indicator; however, most of *Vibrio* patients had presented the cutaneous signs of erythema, swelling and warmth initially at ER, and then quickly developed to serous or hemorrhagic bullae and sepsis few hours later^9,12–15,23^. We found 30 NF patients (63.8%) of non-skin necrosis group developed hemorrhagic or serous bullous skin lesion before emergent surgery, which revealed that the primary pathological site of necrotizing fasciitis affected the deep fascia, and initial cutaneous manifestations did not necessarily reflect the underlying progressive ischemia and destruction^7,10,11^. So we focused on *Vibrio*-related NF patients, who could be observed the clinical course in a short time.

The pathological process of NF is that bacteria proliferate within the superficial fascia and produce enzymes and toxins to spread through the fascia which are diffused inside the arteriolar and capillary vascular systems. Finally, the microorganism proliferation leads to rapid obstruction of the vessels by chemical intimal and subintimal lesions, and epidermal-dermal necrosis may rapidly appear at the skin surface which can quickly progress to hemorrhagic bullae^24–26^. Although skin necrosis and crepitus formation were classified as late stage of NF, and they accounted for 23 to 24.1% of reliable cutaneous signs, which may progress to blisters, serous bullae, and hemorrhagic bullae^10,11^. *Vibrio* species can produce various extracellular toxic factors, such as lipase, protease, enterotoxin, cytolysin, hyaluronidase, and hemolysin, to cause serious collagenolytic, hemorrhagic or edematous skin damage in the extremities by degrading the vascular basement membrane and the type IV collagen^27–29^. We observed that 34.7% (25/72) of *Vibrio* patients had revealed skin necrosis at ER before the definite diagnosis of NF. Thus, we used the skin necrosis as the initial skin sign to diagnose NF at ER.

In this study, the skin necrosis group revealed significantly higher incidences of elevated APACHE score, postoperative intubation, ICU stay, shock at ER, leukopenia, higher banded leukocytes, elevated CRP, and hypoalbuminia than non-skin necrosis group, which indicate that NF patients with initial skin necrosis presentation at ER experienced more fulminant clinical courses and higher mortality rate than those NF patients without skin necrosis. Khamnuan et al. reported they had found 26.7% of NF patients (403/1507) presented skin necrosis, and the appearance of skin necrosis at the time of diagnosis had been identified as a significantly predictive factor for amputation, which the causing pathogens did not include *Vibrio* species in their study^7^. Therefore, we confirm the cutaneous sign of skin necrosis at ER may act as alternative indicator for suspecting NF and predicting the poor prognosis.

Our study needs to be viewed in light of some limitations. First, we assessed only the initial skin condition of *Vibrio* NF patients at the emergency department. Our previous study had reported NF patients with gram-negative bacterial infection were significantly associated hemorrhagic bullae^13^. A further cohort study for investigating the initial skin condition of gram-positive and gram-negative NF may be needed. Second, our study encompassed small sample size and our participants were recruited from only one medical institution. More larger-scale studies are warranted to clarify our viewpoint.

In conclusion, *Vibrio* NF patients presenting skin necrosis at ER were significantly associated with fulminant clinical courses and higher mortality. Physicians should alert the appearance of skin necrosis at ER to early suspect NF and treat aggressively according to those clinical and laboratory risk indicators, such as elevated APACHE score, shock, leukopenia, higher banded leukocytes, elevated CRP, and hypoalbuminia.

## Data Availability

All data generated or analysed are included in this published article s. Further information is available from the corresponding author on reasonable request.

## Abbreviations

NF: Necrotizing fasciitis
ICU: Intensive care unit
ER: Emergency room
APACHE: Acute Physiology and Chronic Health Evaluation
WBC: White blood count
CRP: C-reactive protein
